# Neuromodulation and Eating Disorders

**DOI:** 10.1007/s11920-022-01321-8

**Published:** 2022-02-18

**Authors:** L. Gallop, M. Flynn, I.C. Campbell, U. Schmidt

**Affiliations:** 1 Section of Eating Disorders, Department of Psychological Medicine, Institute of Psychiatry, King’s College London, Psychology & Neuroscience, London, UK; 2 South London and Maudsley NHS Foundation Trust, Maudsley Hospital, London, UK

**Keywords:** Eating disorders, Anorexia nervosa, Neuromodulation, Brain stimulation, Transcranial magnetic stimulation, Transcranial direct current stimulation

## Abstract

**Purpose of Review:**

We review recent evidence on the use of neuromodulation for treating eating disorders (EDs), including anorexia nervosa, bulimia nervosa and binge eating disorder. We evaluate studies on (a) modern non-invasive methods of brain stimulation, such as transcranial magnetic stimulation (rTMS) and transcranial direct current stimulation (tDCS), (b) electroconvulsive therapy (ECT) and (c) more invasive techniques, including deep brain stimulation (DBS).

**Recent Findings:**

Most reports on the clinical applications of neuromodulation in EDs are limited to case studies, case series and small clinical trials. The majority have focused on severe, enduring and hard-to-treat cases of AN. In this population, data suggest that both rTMS and DBS have therapeutic potential and are safe and acceptable.

**Summary:**

High-quality clinical trials in different ED populations are needed which investigate different stimulation methods, sites and parameters, the use of neuromodulation as stand-alone and/or adjunctive treatment, as well as the mechanisms of action.

## Introduction

Eating disorders (EDs) are serious psychiatric disorders with complex biological, psychological and social underpinnings [1]. Anorexia nervosa (AN) is characterised by an intense fear of weight gain and a disturbed body image which motivates severe dietary restriction or other weight loss behaviours, e.g. excessive exercise. Individuals with bulimia nervosa (BN) and binge-eating disorder (BED) experience distressing episodes of objective overeating accompanied by a sense of loss of control, with (in BN) or without (in BED) compensatory behaviours to counteract weight gain (e.g. self-induced vomiting). With mortality rates for AN as high as 5% [2], EDs account for some of the highest rates of morbidity and mortality among mental illnesses.

Clinical guidelines recommend a range of ED focused psychological therapies as first-line treatments for EDs [3]. However, outcomes from the best available psychotherapies for EDs are suboptimal, with remission rates for different EDs ranging between 30 and 60% (e.g. [4]), and little is known about how to proceed when first-line treatments are ineffective. To improve outcomes, novel treatment alternatives are needed.

Neuroimaging techniques have improved our understanding of the neural substrates involved in EDs. Neurobiological models suggest that AN, BN and BED have unique and overlapping neural features, including aberrant functioning of “bottom-up” sub-cortical mesolimbic and reward-related regions and/or in “top-down” prefrontal regions [5, 6]. Atypical functioning of these neural systems is associated with alterations in cognition, reward and emotion, and these may drive and maintain illness related behaviours. These models provide the rationale for investigating and using brain-directed treatments [7].

Here, we provide an update on the literature relating to the use of neuromodulation in EDs in the last 3 to 5 years. Neuromodulation encompasses non-implantable and implantable procedures that can be used to inhibit, stimulate, modify or regulate nervous system functioning for the treatment of disease [8]. This includes techniques ranging from modern non-invasive neurostimulation approaches, such as repetitive transcranial magnetic stimulation (rTMS) and transcranial direct current stimulation (tDCS), to older techniques, such as electroconvulsive therapy (ECT), as well as surgical, invasive methods such as deep brain stimulation (DBS) and vagus nerve stimulation (VNS; see Table 1 for overview of each method). New evidence relating to each neuromodulation technique in EDs is reviewed below.

**Table 1:** Overview of each neuromodulation technique for EDs

**Non-invasive neuromodulation**	**Strengths**	**Weaknesses**
*Repetitive transcranial magnetic stimulation (rTMS)* Electromagnetic coil is used to modulate cortical excitability in a target brain region, e.g. DLPFC	• Minimal adverse effects • Safe and well-tolerated in EDs • Evidence for benefits in BMI, ED and mood symptoms in SE-AN • Evidence for cognitive benefits across EDs	• Time-intensive, 20–30 sessions for optimal effects • Heterogeneity in response • Optimal parameters yet to be determined • Small evidence base in BED and BN • Cost-effectiveness yet to be determined
*Transcranial direct current stimulation (tDCS)* Applies weak direct current to the scalp to modulate existing neuronal activity	• Ease of use and minimal adverse effects • Safe and well-tolerated in EDs • Can be used “online” and applied at-home • Evidence for short-term benefits in cognition and ED symptoms • Low-cost treatment	• Few studies have applied tDCS therapeutically (i.e. > 5 sessions) • Lack of RCTs across EDs
*Transcutaneous auricular vagus nerve stimulation (taVNS)* Attached to the auricular concha and delivers electrical stimulation at the subcutaneous course of the vagus nerve	• Non-invasive alternative to surgical VNS • Can be delivered at-home • Low-cost treatment	• Small evidence base in psychiatric disorders, with only one case series in EDs
*Electroconvulsive therapy (ECT)* Performed under general anaesthesia using electrical stimulation to the scalp to induce a generalised tonic–clonic seizure	• May have positive effects on comorbid mood symptoms •Effect on weight unclear “last resort” treatment option	• Adverse effects, e.g. cognition and memory • Not a long-term treatment option • No RCTs to show clinical efficacy in AN • Not associated with improvement in ED symptoms
**Invasive neuromodulation**		
*Deep brain stimulation (DBS)* Electrical impulses are delivered to illness-relevant brain regions via implanted electrodes	• Preliminary success for improving BMI, ED and affective symptoms in SE-AN • Relatively safe in the short- and long-term • Focal stimulation • Targets subcortical regions that are inaccessible with other neuromodulation techniques	• Adverse side effects (e.g. infection) may be over-represented in SE-AN • Requires neurosurgery • Close monitoring or follow-up is essential which patients may find burdensome • Switching the device off could lead to deterioration of symptoms (i.e. not reversible) • Expensive treatment

### rTMS

Modern non-invasive brain stimulation techniques prominently include rTMS. In rTMS, a current is passed through an electromagnetic coil to induce an increase (high frequency; HF-rTMS) or decrease (low frequency; LF-rTMS) in cortical excitability in target brain regions [9]. Overall, rTMS is well-tolerated and safe [10]. Therapeutic applications of rTMS are being investigated across psychiatric disorders; however, its use is best established in depression where protocols have been incorporated into clinical guidelines [11].

#### rTMS in Anorexia Nervosa

We recently completed the first sham-controlled RCT of high-frequency rTMS applied to the left-DLPFC in 34 cases of severe and enduring anorexia (SE-AN; the TIARA trial [12•]). At 4 months post-randomisation, we saw medium to large between-group effect sizes in mood (e.g. DASS-21, total *d* = 0.9) and quality of life (e.g. EQ-5D-5L, *d* = 0.52) and small between-group differences for BMI and ED symptoms, all favouring real rTMS.

In an open 18-month follow-up of this trial, mood improvements remained broadly stable in the real rTMS group and there was a “catch-up” in the sham group, as 10/12 of these participants subsequently took the opportunity to receive real rTMS treatment [13•]. In regard to BMI change scores, by 18 months, there was a medium between-group effect, along with a higher rate of weight recovery in the real rTMS group compared to sham (BMI above 18.5 kg/m^2^: 46% vs. 9%; *χ*^2^ = 3.67, *p* = .056), suggesting the mood effects of rTMS on AN precede those on BMI.

A notable finding from the participants’ qualitative feedback at the end of TIARA [13•] was a growing flexibility and relaxation around eating and food choices following rTMS. This finding was supported by data from the Food Choice Task (see [14] for task methodology). In the real rTMS group, there was a decrease in self-controlled food choices post-treatment (vs. baseline), reflected in increased selection of tasty-unhealthy foods [15]. This could have arisen because rTMS induced functional changes in the DLPFC and associated neurocircuits: This is partially supported by the arterial spin labelling data obtained in the TIARA study [16]. Unlike previous rTMS studies (e.g. [17]), participants that received real rTMS showed no differences in cerebral blood flow (CBF) at the stimulation site (DLPFC); they did, however, show greater reductions in amygdala CBF, and this was associated with longer-term weight gain [16]. It is possible that lower CBF in the amygdala was associated with reduced fear around food, and this enabled the reported increased flexibility around food post-rTMS (thus resulting in longer-term weight gain). Studies of this potential mechanism underlying weight gain following rTMS are warranted.

The DLPFC is the most widely used target for rTMS in EDs, which may not be optimal for all recipients, e.g. a recent report of rTMS to the left-DLPFC in a case of AN and comorbid depression described no improvement in AN or mood symptoms [18]. As data emerge, new protocols are being developed and alternative stimulatory targets are being examined. For example, a Canadian group targeted the dorsomedial prefrontal cortex (DMPFC) in a case series (*n* = 19; [19]) and reported significant improvements in core symptoms of AN (EDE global score; *p* = .010), anxiety (BAI; *p* < .001) and mood (BDI; *p* = .041) symptoms, from pre- to post-treatment. Their data showed that lower pre-treatment resting state functional connectivity from the DMPFC to the right frontal pole and left angular gyrus correlated with ED symptom improvement. The frontal pole is functionally coactive with the salience network regions (e.g. DMPFC), which have a role in cognitive and impulsive control [20]. Therefore, patients with weaker pre-treatment connectivity between these regions, who also showed greatest improvement in ED symptoms, may be those with reduced capacity for cognitive/impulse control and for whom DMPFC-rTMS may be particularly well-suited. To target different elements of AN (e.g. rigidity), Woodside et al. [19] highlight the potential value of stimulating the right orbitofrontal cortex, which has demonstrated potential for treating obsessive compulsive disorder (OCD) [21].

#### rTMS in BN and BED

Several case studies/series (e.g. [22, 23]) have reported reductions in binge and/or purge episodes in BN following rTMS; however, two RCTs have not found significant differences between real or sham rTMS groups in binge-purge symptoms [24, 25]. An ancillary study to Gay et al. [25] reported on the neurocognitive outcomes [26]: This included inhibitory control (go/no-go task; [27]), impulsivity (Barratt Impulsiveness Scale; [28]), decision-making (Iowa Gambling Task; [29]) and sustained attention (D2 test; [30]) from pre- to post-treatment. Between-group analyses revealed no significant differences in neurocognition post-treatment, but within-group analyses showed improvements in the go/no-go task (*p* = .03) and the BIS cognitive impulsivity subscale (*p* = .01) in the rTMS group, only. On the IGT, the intermediate net score significantly improved (*p* = .002) and a significantly higher proportion of participants understood the task contingencies between pre- and post-treatment after real, but not sham, rTMS. These findings indicate that there are neurocognitive improvements following rTMS but that such changes are not necessarily sufficient to induce clinical improvements in BN [25, 26].

Evidence for the use of rTMS in BED is lacking, although we anticipate the findings of a double-blind, sham-controlled RCT from Maranhão et al. [31].

### tDCS

tDCS involves the application of a constant weak direct current via electrodes placed on the scalp to increase (anodal tDCS) or decrease (cathodal tDCS) cortical excitability [32]. Compared to other neuromodulation techniques, tDCS is easy to use, inexpensive and associated with fewer adverse effects [33, 34].

#### tDCS in Anorexia Nervosa

To date, no RCT has investigated the use of tDCS in AN. A single-blind clinically controlled study in 23 adolescents with AN added either 18 sessions of DLPFC tDCS (anode left/cathode right) (*n* = 12), or family therapy (*n* = 11), to treatment as usual [35]. After 6 weeks of treatment, BMI increased in the tDCS group, an effect that persisted at 1-month follow-up. Participants in both groups showed similar improvements in AN psychopathology, mood and anxiety symptoms. Similarly, significant improvements in AN psychopathology, and a decrease in depression symptoms, were reported in a case series of ten inpatients with SE-AN following 20 sessions of anodal tDCS; however, no changes in BMI were observed [36].

A further case report applied 2 mA anodal tDCS to the left-DLPFC in a case of AN with comorbid PTSD [37]. Improvements were seen in self-reported body dissatisfaction and weight gain, but the onset of type 1 diabetes coincided with the use of tDCS. The effect of tDCS on glucose metabolism remains unclear; however, these findings are not consistent with reports that show reduced blood-glucose in healthy controls following repeated application of tDCS (e.g. [38]).

Finally, an ongoing pilot sham-controlled RCT in Australia [39] is using high-definition tDCS (i.e. more focal stimulation) applied to the left inferior parietal lobule (IPL) in participants with AN. The IPL is a novel neuromodulation target in AN and was selected due to evidence showing reduced connectivity in this region [40] and its role in eye movement, multi-sensory integration and body image. The findings will inform future trials targeting the IPL with tDCS in EDs.

#### tDCS in BN and BED

A single session proof-of-concept RCT showed that tDCS may have potential for treatment of BN [41]. In adults with BED, a sham-controlled crossover trial [42•] investigated the effects of 1 mA (*n* = 16) or 2 mA (*n* = 15) anodal tDCS to the right-DLPFC, vs. sham, on response inhibition in a food-modified antisaccade task. Compared to sham, the 2-mA group improved in response inhibition, reflected in faster latencies of correct antisaccades, whereas the 1-mA group showed slower latencies. Only the 2-mA group showed a significant decrease in self-reported binge eating episodes [42•]. The nonlinear effects of tDCS, such that lower stimulation intensity (i.e. 1 mA) was associated with significantly worse response inhibition, highlight the critical role for stimulation parameters in determining cognitive and/or clinical outcomes.

Our group has recently completed an RCT investigating the use of anodal tDCS to the right-DLPFC combined with approach bias modification (ABM) training for the treatment of adults with BED (see [43] for protocol). ABM training aims to retrain approach bias to appetitive cues into avoidance. Concurrently, administering tDCS is hypothesised to enhance neuroplasticity in pathways activated by the ABM and, in this way, is thought to amplify its efficacy. An integrated qualitative process evaluation [44] suggested that participants experienced ABM training and real/sham tDCS as an acceptable treatment combination, with few side effects; however, clinical outcomes are yet to be published.

An expanding area involves the use of at-home tDCS, which offers several advantages, including ease of access and increased compliance [45]. Several ongoing RCTs are assessing at-home tDCS as a treatment for BED. Our group is administering 10 sessions of concurrent at-home real/sham tDCS and attention bias modification training [46], and Elkfury et al. [47] are combining at-home tDCS with a nutritional counselling therapy, delivering these separately or concurrently in a multi-arm RCT.

#### tDCS in Mixed ED Samples

One study [48] examined the effects of tDCS on implicit preferences for food and body images across EDs (AN *n* = 21; BN *n* = 13; or ED not otherwise specified *n* = 2). Their results showed that anodal tDCS to the medial prefrontal cortex, and the right extrastriate body area, modulated food preferences in EDs. This is relevant as implicit attitudes towards food are reported to predict maintenance of ED symptoms [49]. Future research should assess how these findings replicate in non-mixed ED samples.

### ECT

ECT has long been used to treat mental illness, particularly when a rapid amelioration of severe and/or life-threatening symptoms is needed [50]. However, no randomised controlled trials (RCTs) have examined the efficacy of ECT in EDs. A recent systematic review and single case report by Pacilio et al. [51•] identified 14 cases with EDs treated with ECT from 11 publications. Case reports were mainly limited to AN, apart from one case of BED with comorbid obesity and bipolar disorder. Pre- and post-treatment BMI values were only reported in six cases. Of these, 50% of cases achieved a “normal” BMI over various follow-up periods, including the patient with BED who had a pre-treatment BMI of 97 kg/m^2^ [51•]. The number of ECT sessions was highly variable across case reports (5–45), many patients had significant psychiatric comorbidities, two cases were older adults (> 75 years), and several cases were treated with ECT at a time when specialist ED treatments were not widely available. In their own report of a case with AN and recurrent depression and suicidal ideation, Pacilio et al. [51•] described improvements in mood following ECT, but no improvement in ED symptoms or BMI.

Since then, two further case reports have described the use of ECT in AN in the absence of comorbidities. One case showed gradual weight gain over 12 weeks following ECT [52]; the other case showed no improvement in ED, mood, or anxiety symptoms [53]. It is of note that in both reports where improvements in weight [52] or mood symptoms [51•] were reported, little to no change in attitudes towards weight, shape, or eating was observed. Thus, while the review and case studies available suggest potential value in using ECT to expedite weight restoration in AN [51•], they also highlight the need for adjunctive interventions that address weight and shape concerns. As such, given the evidence in favour of ECT is anecdotal and its safety and tolerability have been questioned, particularly due to the associated memory impairments, we would not presently recommend its use in EDs.

### DBS

DBS is being investigated for its therapeutic potential in EDs, but, as it is an invasive procedure, its use has been limited to those with highly refractory SE-AN. DBS involves surgical implantation of electrodes into key structures (e.g. subcallosal cingulate, SCC [54]) implicated in ED pathology. In a recent randomised, sham-controlled, crossover trial [55•], DBS was applied to two different targets based on comorbidities: the SCC for affective disorders (*n* = 4) and nucleus accumbens (Nacc) for anxiety disorders (*n* = 4). This is relevant given the reported functional connectivity differences between “pure” EDs and EDs with comorbidities [56], as well as AN subtypes [57]. At 6-month follow-up (phase II), there were no significant changes in BMI from baseline to each month post-operatively. Villalba Martínez et al. [55•] surmised that as four participants underwent inpatient treatment to achieve the minimum BMI required for surgery (13 kg/m^2^), the preoperative BMI values did not reflect their “usual” weight. Thus, a BMI reference value (BMI-RV) was calculated from mean BMI in the preoperative period. These findings showed a significant increase (*p* = 0.02) from BMI-RV to BMI at 6-month follow-up, with 5/8 treatment responders (≥ 10% increase in BMI-RV). Phase III of this trial is ongoing, with five responders randomised to one of two arms (DBS switched ON/OFF or OFF/ON) and three non-responders not randomised and continuing with monthly assessments until 12-month follow-up.

The largest case series (*n* = 28) using DBS (to the Nacc) in AN [58] reported significant increases in BMI at 6-month (*p* < .001) and 2-year (*p* < .001) follow-up, with (a) 43% of patients achieving a BMI > 18.5 kg/m^2^ 2 years post-operatively. In addition, obsessive–compulsive, mood and anxiety symptoms significantly decreased from baseline to 6-month and 2-year follow-up. Post hoc analysis revealed that mean BMI post-treatment was significantly lower in the binge-purge subgroup (AN-BP; 16.2 kg/m^2^) than the restrictive subgroup (AN-R; 19.6 kg/m^2^), which might mean that Nacc-DBS is less effective for weight restoration in AN-BP. Of note, the patients were described as “treatment-refractory” simply because many had BMIs < 15 kg/m^2^ and all experienced amenorrhea [58]. However, their illness duration (mean 5.1 years; range 3–10) was markedly lower than other DBS studies in AN (e.g. [59•]) and it seems likely that some of these patients could have recovered with less invasive treatments.

Another group have recently published their ≥ 3-year follow-up data from a case series (*n* = 22) using DBS to the SCC in SE-AN [59•]. Mean BMI increased from baseline (14.0 kg/m^2^) to 1-year (*p* < 0.001; 17.5 kg/m^2^) and 3-year (*p* < 0.003; 16.3 kg/m^2^) follow-up. At the last follow-up (≥ 6 years), 3/15 patients reached BMI ≥ 18.5 kg/m^2^ for ≥ 1 year, and an additional 3/15 reached BMIs between 17 and 18.5 kg/m^2^. After 1-year of SCC-DBS, there was also a significant improvement in obsessive–compulsive, depression and anxiety symptoms that was not maintained at 3-year follow-up. Of note, 20% of the participants experienced hardware infections and 27% had prolonged surgical site pain, which De Vloo et al. [59•] considered may be overrepresented in SE-AN cases compared to other DBS patients. Indeed, a further report [60] described hardware infection, as well as the re-emergence of symptoms, in a case of AN-BP who requested that the stimulation be turned off 19-months postoperatively. The key risk factors for infections following DBS implantation remain largely unclear [61]. Without this information, preventative measures cannot be implemented and the burden of infection (e.g. prolonged antibiotic therapy) may compromise the risk/benefit ratio of this treatment.

Finally, DBS has been applied to the bed nucleus of the stria terminalis in a case of AN-R with significant improvements in weight and binge-purge symptoms [62]. The BNST is thought to be part of the extended amygdala and studies have increasingly used it as the stimulation target in OCD, with encouraging findings (e.g. [63]). Given that AN and OCD share some common neurobiological patterns (e.g. [64]), further examination of the BNST as a stimulation target for DBS in SE-AN seems appropriate.

### VNS

Another invasive neuromodulation technique, VNS is an established treatment for several disorders (e.g. epilepsy), but it has yet to be used in EDs. However, transcutaneous auricular vagus nerve stimulation (taVNS) allows for non-invasive (i.e. without surgery) stimulation of the vagus nerve and is increasingly being studied in psychiatric disorders (see [65] for review). A recent study [66] applied 4 hours of taVNS per day over 9 weeks in a case series of mixed EDs (AN (*n* = 9), BN (*n* = 5), BED (*n* = 1)). Results showed a significant reduction in depression and anxiety symptoms [66]. However, ED symptoms and BMI values were not reported. Therefore, further examination of this procedure, with appropriate and comprehensive clinical outcomes, is warranted.

## Conclusions

In the present review, the majority of studies (~ 80%) have used neuromodulation in AN, with emphasis on rTMS and DBS. For example, findings from the TIARA RCT show that rTMS may be a promising treatment option for patients with SE-AN [12•, 13•]. However, despite encouraging results from rTMS to the DLPFC, outcomes remain heterogeneous, suggesting that DLPFC rTMS is by no means a panacea. Heterogeneity in outcomes may result from neuroanatomical, neurofunctional, connectivity-based, clinical and sociodemographic factors. Indeed, weaker DMPFC-frontal pole connectivity at baseline was associated with greater ED symptom improvement following rTMS to the DMPFC [20] and reductions in amygdala CBF following rTMS to the DLPFC were associated with long-term weight gain in SE-AN [16].

Ultimately, heterogeneity in response will be reduced when we move away from “one-size-fits-all” rTMS protocols for a given disorder and, instead, select optimal parameters based on individual biological, cognitive and clinical markers. This will require trials that incorporate multimodal neuroimaging, plus a range of neurocognitive tasks and clinical measures, as our group did in TIARA. Given the time commitment required, individualising patient care which uses reliable predictors is essential for avoiding arduous yet potentially futile courses of rTMS treatment [67]. Time demands may also be reduced by investigating the relative efficacy of theta burst stimulation (TBS) vs. standard rTMS in EDs, because findings show noninferiority of TBS in depression [68]. TBS is a newer variant of rTMS, but it offers significantly shorter daily stimulation sessions (< 4 mins) compared to standard rTMS (~ 40 mins).

Markedly fewer studies have examined neuromodulation use for BN and BED over the last 5 years. This may be related to negative findings in previous trials (e.g. Walpoth et al. [24]; Gay et al. [25]); however, these were small and had methodological limitations. We therefore encourage further investigation of neuromodulation in BN.

Compared to other neuromodulation approaches, there have been fewer publications of tDCS use in EDs over recent years. Nevertheless, those included in this review paint a promising picture for the positive effects of tDCS on neurocognition, i.e. response inhibition and food preferences [42•, 48]. Cognitive dysfunctions are implicated in the development and maintenance of EDs (e.g. [69]), and thus, improvements in neurocognitive outcomes following tDCS are valuable and may be associated with clinical improvement. It will be of interest to see the outcomes from ongoing trials (e.g. [46, 47]), in particular the use of at-home tDCS in ED’s.

The evidence from recent reports using DBS in SE-AN showed improvements in obsessive–compulsive, depression and anxiety symptoms and BMI, at 1-year [59•] and 2-year [58] follow-ups. However, these were not maintained at ≥ 6-year follow-up in the De Vloo et al. [59•] case series. This may have been due to factors, such as (a) natural disease progression, (b) habituation or (c) placebo effect. If due to (a), this could explain why participants with a markedly lower mean illness duration (5.1 years) maintained significant decreases in clinical symptoms at 2-year follow-up in Liu et al. [58]. Habituation has been reported in other DBS indications (e.g. [70]) and can be mitigated through intermittent, rather than continuous, stimulation, i.e. closed-loop DBS, which was recently used for the first time in a psychiatric disorder [71]. In a case of treatment-resistant depression, Scangos et al. [71] identified amygdala gamma power as a biomarker of depression-specific symptoms for the patient. When prespecified patterns of amygdala gamma power were detected, this triggered 6 s of stimulation to the ventral capsule/ventral striatum. Within 12 days of stimulation, the patient’s symptoms of depression and symptom severity had rapidly improved. Thus, closed-loop DBS presents a potentially exciting avenue for future studies in SE-AN.

Importantly, sham-controlled trials are a necessary next step to quantify the placebo effect of DBS in SE-AN. Though the inclusion of a sham-arm poses a moral/ethical challenge in a neurosurgical context, Villalba Martínez and colleagues [55•] have modelled one solution by using a cross-over sham-controlled design with responders from phase II having DBS turned ON/OFF in phase III. With only five of eight participants qualifying for phase III in this trial, this highlights how carefully patients are selected for trials using DBS. To maximise sample sizes, we recommend that multi-centre clinical trials are employed in the future.

Finally, ECT use has been limited to AN with variable results [51•], while in some cases, it appeared to facilitate weight gain in the short term; this was not true for all cases, and, overall, it did not improve ED symptoms. Given the availability of alternative non-invasive techniques that are not associated with adverse cognitive effects, ECT appears a relatively suboptimal form of therapeutic neuromodulation for treatment of SE-AN.

Overall, while the evidence base suggests that neuromodulation approaches have therapeutic promise in SE-AN and most techniques are safe and well tolerated, there are gaps in the knowledge base. These include questions on the use of neuromodulation treatments in EDs other than SE-AN and issues related to their use as adjunctive versus stand-alone treatments, the relative efficacy of different stimulation methods (e.g. tDCS vs. rTMS), variants (e.g. TBS), stimulation sites and parameters and, lastly, the mechanisms and duration of effects. As we better clarify the neural network disturbances underlying EDs, and how we can use neuromodulation techniques to rectify these, we expect to see increased efficacy.
